# Targeted and systemic therapies for recurrent adult ependymomas: real-world outcomes from a single institution and concise literature review

**DOI:** 10.3389/fonc.2026.1748353

**Published:** 2026-02-18

**Authors:** Marta Maccari, Alberto Bosio, Mario Caccese, Marta Padovan, Angela Guerriero, Giovanni Librizzi, Giovanna Pintacuda, Luisa Bellu, Alba Fiorentino, Francesco Volpin, Tamara Ius, Franco Chioffi, Luca Denaro, Sara Lonardi, Giuseppe Lombardi

**Affiliations:** 1 Department of Surgery, Oncology and Gastroenterology, University of Padova, Padova, Italy; 2 Medical Oncology 1, Veneto Institute of Oncology IOV-IRCCS, Padova, Italy; 3 Department of Surgical Pathology Unit, Padova University Hospital, Padova, Italy; 4 Padova Neuroscience Center (PNC), University of Padova, Padova, Italy; 5 Neuroradiology Unit, Padova University Hospital, Padova, Italy; 6 Radiology Unit, Veneto Institute of Oncology IOV-IRCCS, Padova, Italy; 7 Radiotherapy Unit, IOV-IRCCS Veneto Institute of Oncology, Padova, Italy; 8 Department of Radiation Oncology, General Regional Hospital F. Miulli, (BA), Acquaviva delle Fonti, Italy; 9 Department of Medicine, LUM University, Casamassima, BA, Italy; 10 Division of Neurosurgery, Padova University Hospital, Padova, Italy; 11 Academic Neurosurgery, Department of Neurosciences, University of Padova, Padova, Italy

**Keywords:** bevacizumab, chemotherapy, ependymomas, targeted therapy, temozolomide

## Abstract

**Background:**

Recurrent adult ependymomas lack standard systemic therapies and evidence on chemotherapy and targeted agents is limited. This study aimed to retrospectively evaluated outcomes of systemic therapies, including targeted combinations, in a real-world cohort.

**Methods:**

Adult patients with intracranial or spinal ependymoma treated at our institution between 2013 and 2025 who received at least one systemic line at recurrence were included. Tumor response was assessed according to RANO criteria. Primary endpoints were disease control rate (DCR) and progression-free survival (PFS). Secondary endpoints were overall survival from the start of the first line treatment (OSt) and overall survival from diagnosis (OS).

**Results:**

Among 47 patients, 12 received systemic therapy at recurrence. The median follow up duration for the entire cohort was 28.3 months. Temozolomide (TMZ) was the most commonly used agent (n=12). TMZ monotherapy achieved a DCR of 57% with 6- and 12-month PFS rates of 85.7% and 57.1%, respectively. Targeted therapy was administered to 7 patients: the TMZ-Lapatinib combination provided limited benefit (DCR 33%, median PFS 2.9 months), bevacizumab-based regimens showed variable efficacy; bevacizumab alone achieved a DCR of 33% with one case of prolonged stabilization (>58 months), while bevacizumab plus fotemustine yielded a PFS of 14.1 months. Treatments were generally well tolerated, with limited grade 3 toxicities. Sequential systemic therapy, up to five lines, was feasible in selected cases. Median OSt was not reached; 12-month OS was 66.7%.

**Conclusions:**

This real-world analysis indicates that temozolomide is feasible and associated with disease stabilization in selected patients, while bevacizumab-based combinations showed signals of clinical activity in recurrent adult ependymomas. Prospective, biomarker-driven multicenter trials are warranted to optimize systemic strategies in this rare disease.

## 1 Introduction

Ependymomas are rare central nervous system (CNS) glial tumors that can arise from ependymal cells lining the cerebral ventricles, the central canal of the spinal cord, or cortical rests (1).

According to the Central Brain Tumor Registry of the USA(CBTRUS) the annual incidence of ependymomas is estimated to range from 0.25 to 0.48 per 100.000 persons (2), accounting for 2-3% of all primary CNS tumors and 6.5% of gliomas (3). Ependymomas are relatively more common in children, representing about 5.2% of pediatric CNS tumors, compared to 1.9% in adults. The male-to-female ratio is approximately 1.4:1 (3). Tumor location is age-dependent. In children they predominantly occur intracranially with two third in the posterior fossa, whereas the spinal cord is the most prevalent site in adults (4).

Ependymomas were previously classified according to the WHO 2016 classification into grade I, II and III (or anaplastic) on the basis of pathological criteria alone, while the WHO 2021 identifies 10 subgroups according to a combination of histopathological, molecular and clinical features across three tumor locations (supratentorial, infratentorial and spinal), age group, and tumor grade (5, 6). This integrated approach more accurately reflects the biological and clinical heterogenity of these tumors (7–10). However, this refined classification has not yet translated into the identification of reliable druggable targets (11).

Optimal clinical management of adult ependymomas remains undefined. Most treatment paradigms are derived from pediatric studies, while adult data are limited to small retrospective series. Surgery is the cornerstone of treatment, with gross total resection (GTR) consistently associated with improved prognosis (12–16). The goal is to achieve maximal safe resection without neurological impairment.

Regarding radiotherapy(RT), recent studies have demonstrated the efficacy of local fields irradiation in achieving good local control and low risk of spinal disseminations (17–20). Postoperative radiotherapy is recommended in patients with anaplastic (WHO 2016 grade 3) ependymomas and for those with grade 2 tumors after incomplete resection (17), while the role of postoperative RT in patients with grade 2 ependymoma who undergo complete resection remains controversial (21).

No adjuvant chemotherapy has demonstrated efficacy in randomized studies and systemic therapy is generally reserved for recurrent or progressive disease (22).

According to CBTRUS, the overall relative survival rate is 85.8%, ranging from 91% in patients aged 15–39 to 86.8% in those >40 years (3). Prognosis is influenced by several factors, including extent of resection, tumor grade, location, age <55 years, and good performance status (14, 19, 22, 23).

Given the lack of prospective data, real-world analyses may help define the role of systemic therapy and identify signals of activity worth prospective validation. We therefore conducted a retrospective, single-center analysis of systemic therapies, including targeted treatments, administered at our institution to patients with recurrent ependymomas over the past 10 years. Given the extreme rarity of adult recurrent ependymoma, this study was designed as a descriptive real-world analysis aimed at reporting treatment patterns and clinical outcomes rather than providing comparative efficacy evidence. In addition, we reviewed current literature on conventional and targeted agents to contextualize our findings and explore emerging therapeutic strategies.

## 2 Materials and methods

We conducted a retrospective, single-center study at the Veneto Institute of Oncology (IOV), Padua, Italy, including patients diagnosed with intracranial or spinal ependymoma between April 2013 and April 2025 (data cutoff: April 2025). Clinical records were reviewed to collect demographic and clinical data, including age, sex, tumor location and grade, extent and number of surgical resections, radiotherapy, and systemic treatments.

All patient data were anonymized and stored in an institutional database accessible only to authorized personnel. Histopathological classification followed the WHO classification of CNS tumors in use at the time of the patient’s initial diagnosis. The study was approved by the local ethics committee (IOV-2024-ANIMA).

Patients were eligible for inclusion in the analysis if they had a histologically confirmed diagnosis of ependymoma, had received at least one line of systemic therapy at disease recurrence or progression, and had available follow-up data for outcome assessment. Radiological follow-up was performed with brain and/or spinal MRI every two to three months, or earlier in case of clinical worsening, according to institutional practice. Tumor response and progression were assessed on MRI according to the Response Assessment in Neuro-Oncology (RANO) criteria.

Toxicities associated with systemic treatments were retrospectively extracted from clinical charts and laboratory records, and were graded according to the Common Terminology Criteria for Adverse Events (CTCAE), version 5.0.

The primary endpoints of the study were progression-free survival (PFS), defined as the time from the start of systemic therapy to documented radiological or clinical progression or death from any cause, and disease control rate (DCR), defined as the proportion of patients achieving complete response, partial response, or stable disease as best response to a given systemic therapy. For patients without progression, PFS was censored at the last follow-up or at death in the absence of documented progression. Although systemic treatments and timing varied across the cohort, DCRs were calculated for each regimen for descriptive purposes.

Secondary endpoints included overall survival from first-line treatment (OSt), defined as the time from the start of the first systemic therapy to death or last follow-up and overall survival (OS), defined as the time from initial diagnosis to death or last follow-up.

Descriptive statistics were used to summarize clinical and treatment-related data. Continuous variables were expressed as medians, interquartile ranges, and ranges, whereas categorical variables were reported as counts and percentages. PFS and OS were estimated using the Kaplan–Meier method. Statistical analyses were performed using R software (version 4.4.0; R Foundation for Statistical Computing, Vienna, Austria). Given the limited sample size, all analyses were descriptive and no formal hypothesis testing was performed.

## 3 Results

A total of 47 adult patients diagnosed with intracranial or spinal ependymoma at our institution between 2013 and 2025 were identified. Among them, 12 patients (26%) who received at least one line of systemic therapy for recurrent disease were included in the present analysis. The median follow up duration for the entire cohort was 28.3 months (95% CI, 8.2- 46.2).

The subgroup of 12 patients treated with systemic therapy had a median age at diagnosis of 41 years (range: 18–63), with a slight female predominance (7 females, 5 males). Ten patients had brain tumors (6 supratentorial, 4 posterior fossa) and 2 had spinal lesions. Histologically, 7 patients had WHO grade 3 tumors and 5 had grade 2 at the last surgery. Median Karnofsky Performance Status (KPS) at the time of first line therapy was 70 (range: 60–90). Baseline clinical and treatment characteristics of the 12 patients are summarized in **Table 1**. Most patients (8/12, 67%) presented with neurological symptoms, such as motor deficits (6 patients), dysarthria (2 patients), seizures (1 patient), dizziness (1 patient), cervical pain(1 patient), or peripheral neuropathy (1 patient), while 2 patients were asymptomatic; clinical symptom data were not available or not clearly documented for the remaining two patients. All patients had received surgery and radiotherapy as initial treatment. At recurrence, 5 patients underwent reoperation and 4 received radiosurgery. At recurrence, repeat surgery and/or stereotactic radiotherapy were performed with cytoreductive or local disease control intent. Systemic therapies were initiated only in the presence of residual or progressive disease and were not administered in a true adjuvant or pseudo-adjuvant setting.

**Table 1:** Clinical characteristics of 12 patients with recurrent ependymoma treated with systemic therapies.

Case n.	Sex; age at diagnosis	Grade	Location	Surgery	RT (Gy)	Pattern of PD	KPS	Resurgery (n)	Re-irradiation	Lines of therapy	First Line	Current status	OSt (mo)
1	F;35	G3	supratentorial	STR	PRT	Nodular Leptomeningeal	90	no	no	1	TMZ+CDDP	Dead	0.9
2	F;62	G3	supratentorial	STR	60	Local	70	no	no	1	TMZ	FUP	28.3+
3	M;54	G2	spine C2	STR	SRS	Local	70	no	SRS	1	TMZ	Dead	8.4
4	F;55	G2	posterior fossa	STR	CS	Local	70	1	SRS	3	TMZ	FUP	70.1+
5	F;18	G3	supratentorial	GTR	SRT (21)	Local	70	12	no	5	TMZ DD +LAP	PD	46.2+
6	M;23	G2	spine T4-T10	STR	CS	Local	70	2	no	1	TMZ	FUP	30.6+
7	F;62	G3	posterior fossa	STR	30	Local	70	no	no	3	BEV	Dead	7.9
8	F;63	G2	posterior fossa+ spine C2	STR	PRT	Local	70	no	no	1	TMZ	Treatment ongoing	11.7+
9	M;35	G3	supratentorial	GTR	40	Local	60	4	SRS	1	TMZ	Dead	6.3
10	M;22	G3	supratentorial	STR	50	Local	70	no	no	2	BEV	Lost FUP	8.2
11	M;41	G2	supratentorial	STR	PRT	Local	80	no	SRS	2	TMZ	FUP	24.3
12	F;31	G3	supratentorial	STR	50	Local	80	6	no	1	TMZ+CDDP	Dead	12.9

PD, Progression; KPS, Karnofsky Performance Status; GTR, gross total resection; STR, subtotal resection; CS, craniospinal radiotherapy; PRT, proton therapy; SRT, stereotactic radiotherapy; SRS, radiosurgery; TMZ, temozolomide; BEV, bevacizumab; TMZ DD, temozolomide dose dense; LAP, lapatinib; CIS, cisplatin; FUP, follow up; LOST FUP, lost to follow up; OSt, time from start of systemic therapy to death or last follow-up; mo, months; “+” indicates censored observation (alive at last follow-up).

Median time to first-line therapy (mTTT), defined as the time from initial diagnosis to the start of the first systemic therapy, was 37.8 months (IC 95%: 19,5 – 128,5; IQR 19.77-108.90, range 6.8-287.0).

The number of systemic therapy lines ranged from 1 to 5 (median 2). Details of systemic therapies administered and related outcomes are summarized in **Table 2**.

**Table 2:** Summary of systemic treatment regimens and associated outcomes.

Therapy	Schedule	No of pts	Median number of cycles (range)	mPFS (mo) [95% CI]	range	PFS-6 (%) [95% CI]	PFS-12(%) [95% CI]	DCR
TMZ	Temozolomide 200 mg/mq day 1–5 q4w	7	12 (1-14)	NR (7.0–NE)	2.7–25.3	85.7 (63.3–100)	57.1 (30.1–100)	57% (1 PR)
TMZ + Cisplatin	Temozolomide 150 mg/mq day1-5 + Cisplatin 75 mg/mq q3w	3	2(1-9)	0.9 (0.8–NE)	0.8–9.1	33 (6.7-100)	0	33% (1 SD)
TMZ DD+ Lapatinib	Temozolomide DD 125mg/mq day 1-7; 15–21 q4w +Lapatinib 1250mg/unit	3	2 (1-5)	2.9 (1.2–NE)	1.2–5.9	0	0	33% (1 SD)
TMZ +bevacizumab	Temozolomide 200 mg/mq day 1–5 q4w + Bevacizumab 5mg/Kg	2	8(2-14)	9.5 (3.3–15.7)	3.3–15.7	50 (12.5–100)	50 (12.5–100)	0
Bevacizumab alone	Bevacizumab 10 mg/kg q2w	3	5(3-37)	1.8 (1.6–NE)	1.6–21.0	33 (6.73-100)	33 (6.7-100)	33% (1SD>3yrs)
Bevacizumab+Fotemustine	Bevacizumab 5 mg/kg + Fotemustine 80mg/mq q2w	1	28	14.1 (single pt)	–	100	100	(SD>1yrs)
Fotemustine	Fotemustine 80mg/mq q2w for 5 cycles → q4w	1	5	2.40 (single pt)	–	0	0	100% (SD)
Lomustine +Procarbazine	Lomustine (CCNU) 110 mg/m², day 1, q6w Procarbazine 60 mg/m², days 8–21, q6w	1	2	3.22 (single pt)	–	0	0	0

TMZ, Temozolomide; TMZ DD, Temozolomide dose dense; mo, months; yrs, years, pt, patient; NR, not reached; NE, not estimable; DCR, disease control rate; PFS, progression free survival; PFS-6-12, PFS rates at 6 and 12 months. SD, stable disease; PR, partial response.

Temozolomide (TMZ) was the most frequently used agent, administered either as monotherapy (7 patients) or in combination (8 patients). Combination regimens included TMZ with cisplatin (3 patients), TMZ with lapatinib (3 patients), TMZ with bevacizumab (2 patients) and lomustine with procarbazine (1 patient). Other agents included fotemustine (1 patient) and bevacizumab, given either alone (3 patients) or combined (1 patient). The most common first-line regimen was single-agent TMZ.

**Figure 1:**
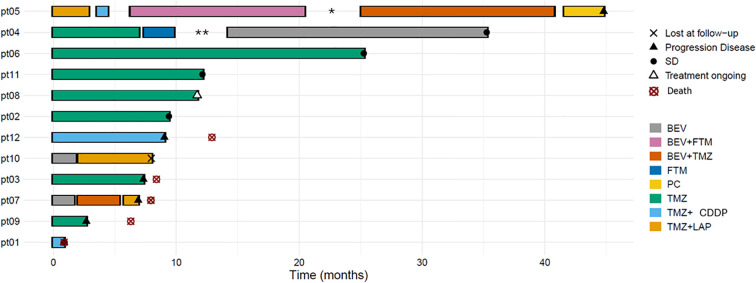
Timeline of systemic treatments for the 12 patients. Each horizontal bar represents the duration of a systemic therapy. Time is expressed in months from the start of systemic therapy. Different colors correspond to treatment regimens. BEV, bevacizumab; BEV+FTM, bevacizumab + fotemustine; BEV+TMZ=bevacizumab + temozolomide; FTM, fotemustine; PC, lomustine+procarbazine; TMZ, temozolomide; TMZ+CDDP, temozolomide + cisplatin, TMZ+LAP, temozolomide dose dense + lapatinib. Symbols indicate clinical events as reported in the legend × = lost to follow-up; ▴ = progressive disease; • = stable disease (SD); △ = treatment ongoing; ⊗ = death. * patient underwent re-operation, ** patient underwent radiosurgery.

Regardless of the line of therapy, the median progression-free survival (mPFS), progression-free survival rates at 6 and 12 months, and disease control rate (DCR) for each regimen were as follows. Temozolomide (TMZ) monotherapy yielded a mPFS that was not reached (95% CI, 6.9–not estimable; range, 2.7–25.3), with PFS rates of 85.7% (95% CI, 63.3–100) at 6 months and 57.1% (95% CI, 30.1–100) at 12 months, and a DCR of 57% (including one partial response). The combination of TMZ plus cisplatin was associated with a mPFS of 0.89 months (95% CI, 0.8–not estimable; range, 0.8–9.0), with PFS rates of 33% (95% CI, 6.73–100) at 6 months and 0% at 12 months, and a DCR of 33%. TMZ plus lapatinib showed a mPFS of 2.9 months (95% CI, 1.15–not estimable; range, 1.2–5.9), with PFS rates of 0% at 6 and 12 months, respectively, and a DCR of 33%. Bevacizumab monotherapy achieved a mPFS of 1.8 months (95% CI, 1.6–not estimable; range, 1.6–21.0), with PFS rates of 33% (95% CI, 6.7–100) at both 6 and 12 months, and a DCR of 33%, including one patient who achieved stable disease during treatment and remained progression-free at last follow-up (PFS: 58.0 months). The combination of bevacizumab and fotemustine in one patient led to a PFS of 14.1 months with SD as best response. Fotemustine monotherapy resulted in a PFS of 2.4. Bevacizumab plus TMZ showed a mPFS of 9.5 months (95% CI, 3.3–15.7; range, 3.2–15.7), with PFS rates of 50% (95% CI, 0–100) at both 6 and 12 months. Finally, lomustine plus procarbazine (PC) was associated with a PFS of 3.2 months (1 patient). **Figure 1** summarizes the timeline and sequence of systemic treatments administered to the 12 patients, with duration and clinical outcomes indicated for each line of therapy.

At the data cut-off in April 2025, six patients were alive: four were in follow-up after stable disease, and two remained on active treatment. At last follow-up, three patients treated with first-line temozolomide monotherapy remained in stable disease despite treatment discontinuation, reflecting prolonged disease stabilization rather than ongoing active treatment.

Median overall survival from the start of systemic therapy was not reached at the time of analysis. The overall survival rate from the start of systemic therapy rates at 6, 12, and 24 months were 91.7% (95% CI, 53.9–98.8), 66.7% (95% CI, 33.7–86.0), and 58.3% (95% CI, 27.0–80.1), respectively. Median overall survival from diagnosis was 180.9 months (95% CI 17.8-NE). The overall survival from diagnosis rates at 6, 12, and 24 months were 100.0% (95% CI, 100.0–100.0), 91.7% (95% CI, 53.9–98.8), and 83.3% (95% CI, 48.2–95.6), respectively(see **Supplementary Figures S1**, **S2**).

Systemic treatments were generally well tolerated. Hematologic toxicities were the most frequent: thrombocytopenia occurred in 5 patients (including one grade 3), and neutropenia in 2 (both grade 2), mainly during TMZ-based treatments. One case of grade 1 transaminase elevation was reported with TMZ. Bevacizumab was associated with grade 1 hypertension in one case, and grade 2 nausea was observed in a patient treated with TMZ plus cisplatin. No grade ≥4 toxicities or treatment discontinuations occurred. Treatment discontinuation in patients achieving stable disease was mainly driven by real-world clinical decision-making, including patient preference, treatment fatigue and prolonged disease stability, rather than treatment-related toxicity.

## 4 Discussion

Recurrent adult ependymomas remain a clinical challenge due to their rarity, biological heterogeneity, and the absence of standardized systemic treatments. Most of the available data come from pediatric populations or small retrospective series, and prospective evidence in adults remains scarce. **Table 3A** and **3B** summarizes the main studies evaluating chemotherapy and targeted agents for recurrent ependymoma.

Table 3ASummary of published studies on systemic therapies for recurrent ependymoma.

**Table:** 

Study (year)	Therapy	Type	Setting		N pts	Grade(%)	RR(%)	mPFS (mo)	mOS	
Chemotherapy										
Rudà (24) 2016	TMZ	Retrospective		WHO G2 or 3 failing re-operation and/reirradiation	18	II(45) III(55)	CR(5) PR(17)	9.7		30.5
Chamberlain (25) 2009	TMZ	Retrospective		WHO G2 platinum refractory	25	II(100)	CR(0) PR(4)	2		3
Gilbert (27) 2021	Dose dense TMZ + Lapatinib	Phase II Single arm		Recurrent intracranial and spinal	50	I(16) II(32) III(40)	CR(4) PR(12)	7.8		27
Brandes (26) 2005	Cisplatin	Retrospective		Cisplatin-based(46%) vs non cisplatin based (54%)	13 vs 15	II(61) III(39)	CR(15,4)vs (0) PR(15,5) vs (13,3)	9.9 vs 10.9		40.7
Gornet (28) 1999	Platinum-based	Retrospective		Platinum based vs nistrosourea based	6 vs 8	II(50) III(37)	PR/MR(67)vs(25)	6 vs 10		–
Other therapies										

Study CT identifier (year)	Therapy	Type		Setting	N pts	Schedule	RR(%)	Results		
NCT01032070 2016	Erlotinib	Randomized open label Phase II		Recurrent or Refractory pediatric ependymoma	25	Erlotinib 85 mg/m2 daily vs Etoposide 50 mg/m2 daily 3 weeks q4w	CR(0) PR(0) SD(15,4) vs CR(0) PR(16,7) MR(8,3) SD(16,7)	mPFS 52 vs 65 days		
NCT01462695 2016	Sunitinib	Open label Single arm Phase II		Recurrent or Refractory pediatric ependymoma/ HGG	30	Sunitinib 15 mg/m2–4 weeks q6w	No ORR	mPFS 83 days		
NCT02155920 2023	Everolimus	Open label Single arm Phase II		Recurrent or Refractory pediatric posterior fossa and spinal ependymoma	11	Everolimus 4.5 mg/m2 daily q4w	No ORR	mPFS 48days		
Green (31) 2009	Bevacizumab	Retrospective		Recurrent adult ependymoma	8	Bevacizumab10 mg/Kg iv q2w alone or in combination	PR(75) 1 SD >8months	mTTP 6.4 months mOS 9.4		
NCT00883688 2015	Bevacizumab + Lapatinib	Phase II		Recurrent Pediatric ependymoma	24	Bevacizumab 10 mg/kg iv days q2w + lapatinib 900mg/m2	No ORR Prolonged SD in 4pts	mTTP 7.9 weeks		
NCT02054806 (Keynote 028)	Imetelstat	Molecular biology Phase II		Recurrent medulloblastoma, HGG, ependymoma candidate to surgery	42 (4 ependymomas)	Imetelstat 2-h iv at 285 mg/m2 12–24 h before surgery.	No ORR Intratumoral and PBMC target inhibition	interrupted for toxicity		
NCT02940483	5-Azacytidine	Phase 0		Recurrent ependymoma udergoing maximal safe resection	6	5-AZA 10 mg into the fourth ventricle q1w, 12 cycles	No ORR 2pts reduction size one ventricular lesion	No AE		
NCT04958486	5 Azacitidine + Trastuzumab	Early Phase I		Recurrent adult and pediatric with Recurrent or Residual Posterior Fossa Ependymoma	4	5 AZA + Trastuzumab into fourth ventricle	NA	NA completed, no results available yet		
NCT03434262	Ribociclib + Gemcitabine	Phase I		Recurrent brain tumors	11 Ependymomas	Ribociclb+ combination molecular driven therapy	NA	PFS>2 yrs in 1 pt		

CT, clinical trial; TMZ, temozolomide; mPFS, median progression free survival; mOS, median overall survival; TTP, time to progression; RR, Response Rate; ORR, objective response rate; CR, complete response; PR, partial response; MR, minimal response; SD, stable disease; pts, patients;q1w, weekly; q2w, every 2 weeks; q3w, every 3 weeks;q4w, every 4 weeks; HGG, high grade gliomas; iv, intravenous; icv, intraventricular; AZA, azacitidine; AE, adverse event; NA, not available.

**Table 3b:** Ongoing clinical trials for recurrent ependymoma.

Study CT identifier (year)	Drug	Type	Design	Population	Status
NCT04661384	IL13Ralpha2-CAR T cells	Phase I	IL13Ralpha2-CAR T Cells icv delivery on day 1 qw for 4 cycles as adjuvant therapy	Leptomeningeal disease from glioblastoma, ependymoma, medulloblastoma	Active not recruiting
NCT02774421	Trastuzumab	Phase I	Intrathecal trastuzumab + subcutaneus GM-CSF	Pediatric recurrent posterior fossa ependymoma	Active not recruiting
NCT03033992	TTF	Not applicable	Optune ≥ 18 hours/day for at least 23 days out of 28 days of cycle one	Pediatric recurrent/refractorysupratentorial glioma or ependymoma	Recruiting
NCT01795313	Peptide vaccine + Imiquimod	Phase I	HLA-A2 restricted tumor antigen vaccine+	Recurrent pediatric Ependymoma	Recruiting

GM-CSF, granulocyte-macrophage colony-stimulating factor, HLA, human leukocyte antigen, icv, intraventricular, TTF, tumor treating fields.

In this context, our mono-institutional retrospective analysis of 12 adult patients with recurrent intracranial or spinal ependymomas treated with systemic therapies over a 10-year period provides clinically relevant insights.

Temozolomide (TMZ) was the most frequently administered agent, either as monotherapy or in combination. In our cohort, TMZ monotherapy achieved a disease control rate (DCR) of 57% and although the median PFS for temozolomide was not reached by Kaplan–Meier estimation, the observed median follow-up time was 9.4 months, which aligns with findings by Rudà et al. (24), who reported a 22% objective response rate (ORR) and a mPFS of 9.7 months in a retrospective study of 18 adult patients. The observation of prolonged stable disease in a subset of patients treated with temozolomide should be interpreted cautiously, as this reflects disease control in a real-world setting rather than durable objective radiological responses. Conversely, Chamberlain et al. (25) observed limited efficacy of TMZ in platinum-refractory recurrent ependymomas (ORR 4%, mPFS 2 months), suggesting that TMZ is more effective when used earlier in the treatment course and in patients not heavily pretreated.

TMZ combinations provided more modest benefit. TMZ plus cisplatin resulted in a DCR of 33% and mPFS of 0.9 in our series, consistent with literature data indicating limited additional efficacy from platinum-based regimens in adults (26). Interestingly, fotemustine, a nitrosourea similar to lomustine with high CNS penetration, showed promising activity in combination with bevacizumab (PFS 14.1 months) in a heavily pretreated patient, supporting further exploration of this agent.

The combination of TMZ and lapatinib, a dual EGFR/HER2 tyrosine kinase inhibitor, yielded a DCR of 33% and mPFS of 2.9 months in our study. These outcomes were inferior to those reported in a phase II trial conducted by the Collaborative Ependymoma Research Network (CERN), where the dose-dense TMZ plus lapatinib regimen achieved a 16% ORR and mPFS of 7.8 months in 50 adult patients (27). One potential explanation is the lack of molecular selection in our cohort. In the CERN study, patients with high ErbB2 (HER2) expression appeared to derive greater benefit. Therefore, incorporating molecular profiling, such as EGFR and HER2 status, may optimize patient selection for lapatinib-based treatments.

Among targeted therapies, bevacizumab demonstrated noteworthy activity in selected patients. Green et al. (28) reported a 75% ORR and mPFS of 6.4 months in a small adult cohort. Our findings support this observation: one patient achieved 21 months of disease stabilization on bevacizumab monotherapy and remains progression-free 58 months after treatment initiation, despite discontinuation of therapy. Furthermore, the most durable stabilization in our series was achieved in a heavily pretreated patient receiving sequential bevacizumab-containing regimens (bevacizumab plus fotemustine with PFS of 14.13 followed by bevacizumab plus temozolomide with a PFS of 15.67), supporting the potential benefit of bevacizumab-based combinations in this setting. While such outlier responses are rare, they highlight the need to identify predictive markers (e.g., VEGF expression, radiologic features) to select patients more likely to benefit from anti-angiogenic therapy. Conversely, in pediatric trials (29) bevacizumab failed to induce objective responses, though some patients experienced prolonged stable disease.

Overall, our data suggest that a subset of adult patients may benefit from targeted treatments. While lapatinib’s efficacy may be limited without patient enrichment strategies, its safety profile and blood-brain barrier permeability justify further investigation in biomarker-driven trials. Bevacizumab, although not universally effective, may provide durable control in angiogenesis-dependent tumors, potentially in combination with nitrosoureas. Notably, the combination of bevacizumab and fotemustine was evaluated for the first time in this pathology in our study, showing encouraging disease stabilization.

Another relevant finding from our series is the feasibility of multiple lines of systemic therapy. The number of lines ranged from 1 to 5 (median 2), with some patients achieving prolonged overall survival (OS) beyond 8 years from diagnosis. This challenges the notion that recurrent ependymomas are uniformly aggressive and rapidly fatal. In patients who maintain good clinical condition over time, the use of sequential systemic therapies may contribute to prolonged survival. Although no specific regimen emerged as clearly superior, switching between different chemotherapy classes (e.g., alkylators, nitrosoureas, anti-angiogenics) appears reasonable, given the heterogeneity of treatment response and lack of cross-resistance. This approach is supported by retrospective studies (30) who reported that various chemotherapies, including temozolomide, nitrosoureas, and platinum compounds, achieved disease stabilization in a significant proportion of adult patients with intracranial ependymoma, even across multiple treatment lines, thus supporting the feasibility and potential value of sequential therapeutic strategies.

Regarding toxicity, systemic therapies were generally well tolerated. TMZ was associated with manageable hematologic toxicity (thrombocytopenia in 5 patients, including one grade 3; neutropenia in 2 patients, both grade 2). Bevacizumab led to grade 1 hypertension in one case, and no thromboembolic or hemorrhagic complications were observed. Lapatinib did not result in clinically significant adverse events in our cohort, likely due to limited exposure. These findings mirror safety data from the CERN study (27), where the combination of TMZ and lapatinib was well tolerated, with fatigue, nausea, and diarrhea being the most common side effects.

Nevertheless, this study has some limitations. First, its retrospective and single-center design limits the generalizability of the findings and may introduce selection biases and confidence intervals for PFS and OS were wide, reflecting the small cohort size rather than uncertainty in data measurement. Second, the small sample size precludes definitive conclusions on comparative efficacy across treatment regimens.

Third, an important limitation of this study is the lack of systematic molecular profiling according to the WHO 2021 classification, which reflects the retrospective real-world nature of our cohort and the long time span of patient inclusion. In particular, molecular subgrouping and target expression analyses were not consistently available, limiting biologically driven treatment selection.

Although available prospective data on lapatinib in recurrent ependymoma were not primarily biomarker-driven, the CERN phase II study suggested that patients with higher ErbB2 (HER2) expression might derive greater clinical benefit (27). Therefore, the absence of EGFR and HER2 assessment in our cohort may have limited the ability to optimize patient selection for lapatinib-based therapies and should be considered when interpreting the observed outcomes. Similarly, no validated predictive biomarkers have been identified for bevacizumab activity in ependymoma, and its clinical use remains mainly guided by radiological and symptomatic features rather than molecular characteristics. In addition, MGMT promoter methylation status was not routinely assessed in this retrospective cohort and was therefore unavailable for outcome correlation; unlike glioblastoma, the predictive role of MGMT methylation for temozolomide response in ependymoma remains controversial and poorly validated, further limiting biomarker-based treatment stratification. These limitations should be taken into account when interpreting the efficacy signals observed in this real-world series.

Fourth, given the rarity of adult recurrent ependymoma, several treatment regimens in our cohort were administered to very small patient subgroups. Therefore, efficacy outcomes observed in subgroups including one to three patients should be interpreted as purely descriptive and hypothesis-generating, particularly in the presence of wide confidence intervals. These findings should not be considered comparative or definitive evidence of treatment superiority.

Yet, both spinal and intracranial ependymomas were included, which, despite reflecting real-world heterogeneity, may confound interpretation due to their distinct biological behavior. Radiological assessments were also conducted retrospectively without central review, which may have affected consistency in response evaluation. Furthermore, the same treatment was administered at different lines of therapy across patients, which may have influenced the evaluation of its efficacy, as treatment effectiveness can vary depending on timing and prior exposure.

Despite these limitations, our analysis provides clinically relevant, real-world insights into the use of systemic therapies for recurrent adult ependymomas, a rare disease where prospective data are lacking.

In our real-world experience, temozolomide appeared to be feasible and was associated with disease stabilization in selected patients. However, conclusions regarding consistent antitumor efficacy remain limited by the retrospective design and small sample size, while bevacizumab-based strategies and molecularly informed treatments warrant further exploration in prospective multicenter settings. The possibility of administering multiple systemic lines offers a window of opportunity to prolong survival in fit patients.

Future research should prioritize the integration of molecular profiling to better guide patient selection for targeted agents, and the design of prospective, multicenter trials specifically dedicated to adults. Novel approaches such as immunotherapy, tumor treating fields, and CAR-T cell strategies are currently under investigation reflecting the growing interest in immuno- and device-based modalities for this rare disease and may offer additional options in the recurrent setting(see **Table 3b**). Finally, collaborative real-world registries will be instrumental in defining treatment sequencing and improving outcomes in this rare disease.

## Data Availability

The original contributions presented in the study are included in the article/**Supplementary Material**. Further inquiries can be directed to the corresponding author.
