# Supplementary material for: Targeted and systemic therapies for recurrent adult ependymomas: real-world outcomes from a single institution and concise literature review
Source: Front Oncol. 2026 Feb 18;16:1748353. doi: 10.3389/fonc.2026.1748353 (PMC12956718; doi:10.3389/fonc.2026.1748353)
Supplement: Supplementary file 1 [file DataSheet1.docx]

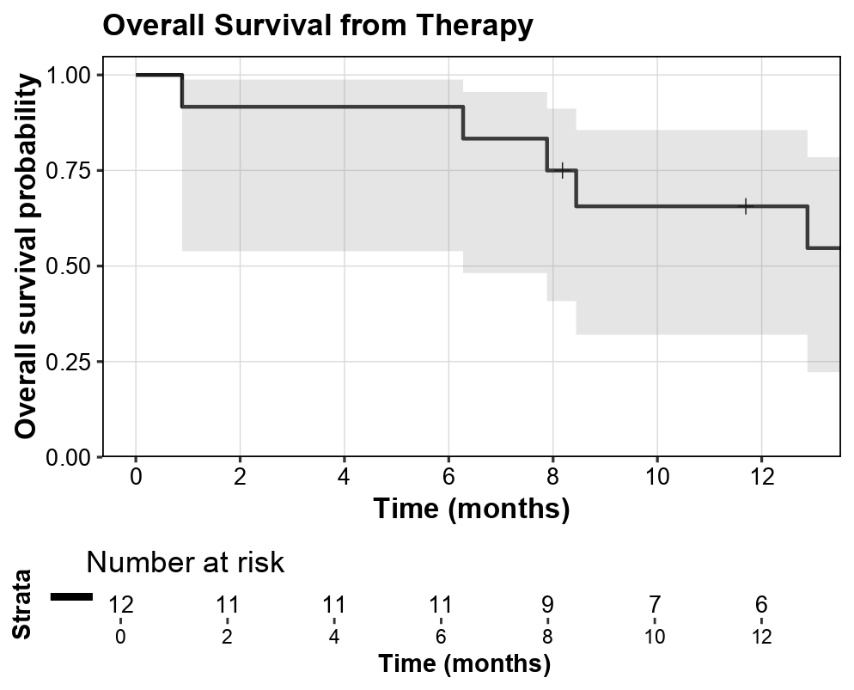
**Figure S1.** Overall Survival from Therapy

**Figure S1**. Kaplan–Meier curve showing overall survival probability from the date of diagnosis in the study cohort (n = 12). The shaded area represents the 95% confidence interval. Vertical tick marks indicate censored cases.

**Figure S2.** Overall Survival from Diagnosis


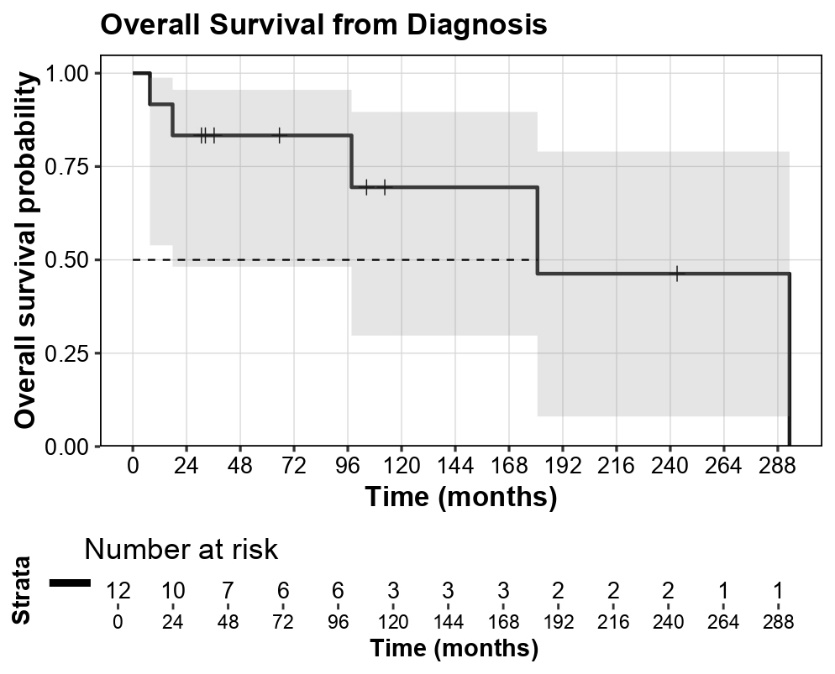


**Figure S2**. Kaplan–Meier curve showing overall survival probability from the start of systemic therapy (n = 12). The shaded area represents the 95% confidence interval. Vertical tick marks indicate censored cases.
